# *Magnolia officinalis* Bark Extract Prevents Enterocyte Death in a Colitis Mouse Model by Inhibiting ROS-Mediated Necroptosis

**DOI:** 10.3390/antiox11122435

**Published:** 2022-12-09

**Authors:** Kang-In Lee, Hye Jin Kim, Hyungjun Kim, Min-Soo Kim, Jung Im Kim, Ki-Sun Park

**Affiliations:** 1 KM Science Research Division, Republic of Korea Institute of Oriental Medicine, Daejeon 34054, Republic of Korea; 2 KM Convergence Research Division, Republic of Korea Institute of Oriental Medicine, Daejeon 34054, Republic of Korea

**Keywords:** necroptosis, inflammatory bowel syndrome, colitis, *Magnolia officinalis*, reactive oxygen species

## Abstract

Necroptosis is a form of programmed cell death with features of necrosis and apoptosis that occurs in the intestinal epithelium of patients with inflammatory bowel disease (IBD), including ulcerative colitis and Crohn’s disease. In addition, necroptosis has also been observed in enterocytes in animal models of dextran sulfate sodium (DSS)-induced colitis. Thus, the discovery of natural products for regulating necroptosis may represent an important therapeutic strategy for improving IBD. We found that *Magnolia officinalis* bark extract (MBE) prevented weight loss and suppressed the activation of the proinflammatory cytokine IL6 in DSS-induced colitis. Furthermore, MBE restored the length of the damaged colon and decreased the expression of necroptosis markers in mice with DSS-induced colitis. In vitro, necroptosis-induced reactive oxygen species (ROS) production was reduced by MBE, and the expression of COX2, a target protein of ROS, was simultaneously suppressed. Both magnolol and honokiol, the two major bioactive compounds in MBE, inhibited necroptosis in human primary intestinal epithelial cells and colorectal adenocarcinoma cells. Our findings highlight the effectiveness of MBE in modulating enterocyte necroptosis and suggest that MBE may be developed as a natural, disease-targeting drug for the treatment of colitis.

## 1. Introduction

Inflammatory bowel disease (IBD) is prevalent worldwide, affecting 1.5 million people in North America and 2 million people in Europe [1]. IBD is commonly referred to as an intestinal disease and includes ulcerative colitis and Crohn’s disease. IBD was on the rise in the Western World until the 20th century, but in the 21st century, its occurrence rate has increased rapidly in regions such as South America, Eastern Europe, Asia, and Africa [2]. Dietary habits, aging, genetic factors, and homeostatic disruption of the immune system are among the primary causes of IBD, which together result in a markedly diminished ability of most IBD patients to combat chronic inflammatory responses [3,4,5]. Chronic inflammatory responses disrupt the tight junctions between intestinal epithelial cells and gut microorganisms, allowing their products to migrate to the lamina propria [6,7]. Lymphocytes, macrophages, eosinophilic leukocytes, and mast cells present in the lamina propria become activated and secrete inflammation-related cytokines and chemokines to activate the mechanisms that promote epithelial cell death. Ultimately, intestinal pathogen invasion is further accelerated and exacerbated by positive feedback, followed by an inflammatory response [8,9].

Enterocytes, which constitute the gut epithelium, are the primary barrier preventing the penetration of pathogens into the intestine [10,11]. Recent studies have reported that enterocyte death due to acute and chronic inflammation is closely linked to the necroptotic pathway [12,13]. Necroptosis induces cell death through programmed cell death (PCD) mechanisms similar to apoptosis; however, the PCD mechanism is distinguished from apoptosis in that it occurs independently of the caspase 8-dependent pathway [14]. Evidence suggests that necroptosis is initiated by the activation of the fas cell surface death receptor (FAS), lipopolysaccharide (LPS), and tumor necroptosis factor-related apoptosis-inducing ligand (TRAIL) receptors, which sequentially induce phosphorylation of RIP1, receptor-interacting protein kinase3 (RIPK3 or RIP3), and mixed lineage kinase domain-like pseudokinase (MLKL) (necrosome) [15,16]. Finally, phosphorylated MLKL (pMLKL) moves to the inner plasma membrane, causing cell swelling and membrane rupture [17,18]. In a recent study, necroptosis was observed in patients with ulcerative colitis and in animal models of dextran sulfate sodium (DSS)-induced colitis [19,20,21]. Lee et al. confirmed a significant increase in RIPK3 and MLKL in ulcerative colitis patient tissues, and treatment with the RIPK3 inhibitor, GSK’872, in peripheral blood mononuclear cells isolated from the patient suppressed the activated inflammatory response [22]. Treatment with GSK’872 also reduced the inflammatory response in a DSS-induced colitis animal model, resulting in the recovery of damaged enterocytes [23]. Chong et al. found that expression of the HtrA2 gene was significantly decreased along with the activity of pMLKL in mice treated with DSS for seven days. The authors developed a strategy to selectively inhibit necroptosis using UCF-101, a serine protease inhibitor of HtrA2, and found UCF-101 to be a potential anti-colitis therapeutic [19]. Therefore, discovering novel substances that inhibit RIPK3 and MLKL, while understanding the mechanism of necroptosis in intestinal diseases, may be an excellent strategy for developing treatments for IBD.

*Magnolia officinalis* bark extract (MBE) has been used as a traditional medicine for over 2500 years in East Asian countries, such as China, Japan, and Republic of Korea [24]. MBE is composed of various phenolic compounds, with magnolol (MN) and honokiol (HK), which are classified as lignans, being the major bioactive compounds [25]; MN and HK inhibit carcinogenic processes in breast cancer, melanoma, and head and neck squamous cancer. In addition, MBE is effective for the treatment of asthma, diabetes, and neuronal diseases, as well as for improving the digestive system, including the regulation of gastrointestinal (GI) motility, antimetric and antidiarrheal effects, antiulcer effects, and improvement of GI dysfunction [26]. MN and HK have inhibitory effects on tumor necrosis factor-alpha (TNF-α), prostaglandin E2 (PGE2), and nitric oxide (NO) production; inhibit the activity of nuclear factor kappa B (NFκB), a redox-sensitive transcription factor; provide antioxidant effects through the inhibition of ROS; and can reduce allergy symptoms by inhibiting histamine release [26]. These mechanisms are closely related to the mechanism of cyclooxygenase 2 (COX2) inhibition, which is a factor related to immune cell inflammation in the context of diseased intestinal cells both in vivo and in vitro, as well as the mechanism inhibiting the release of histamine from immune cells [27,28,29]. Nevertheless, the potential roles of MBE in necroptosis regulation and IBD alleviation are still unknown.

Using the DSS-induced colitis mouse model, we confirmed a significant increase in necroptosis signals in the large intestine and observed that MBE reduced the expression of necroptosis markers in a dose-dependent manner. Furthermore, the ROS-induced increase in COX2 expression was significantly reversed by MN and HK. Mechanistically, MBE represses RIPK3 and MLKL by modulating JNK/p38-MAPK/COX2 signaling induced by ROS. Our study supports the further investigation of MBE as a potential natural therapeutic product for ameliorating IBD.

## 2. Materials and Methods

### 2.1. Animals and In Vivo Experiments

**Figure 1:**
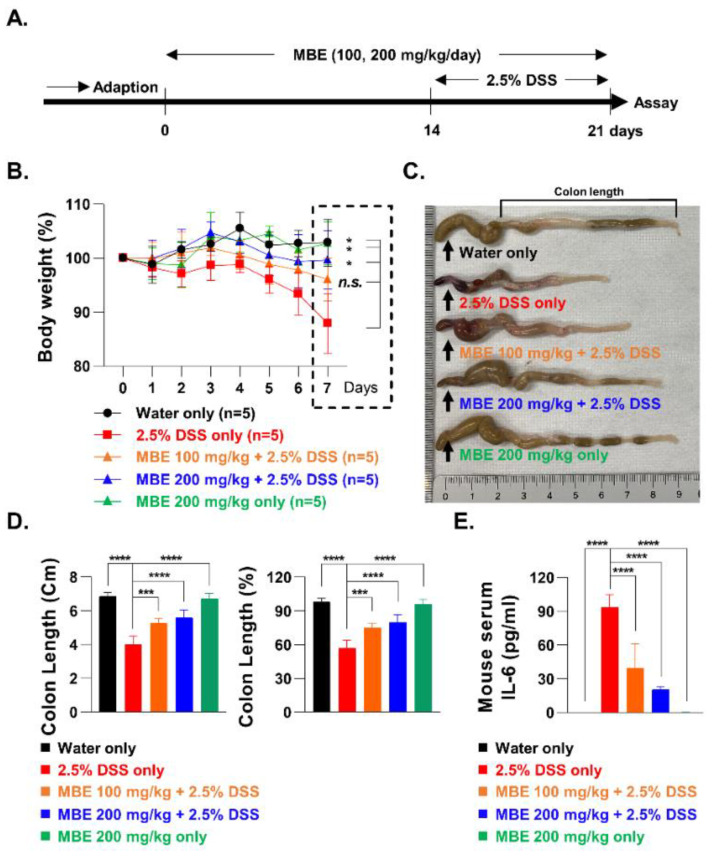
MBE treatment alleviates DSS-induced colitis. (**A**) Schematic representation of MBE oral administration to mice. (**B**) The weights of mice were recorded daily for the last 7 days of treatment. (**C**) Representative images of the colon length from each group. (**D**) All colon lengths were measured from the ileocecal junction to the distal end of the rectum. (**E**) IL-6 levels in mouse serum were measured using ELISA. *n* = 5 for each group. * *p* < 0.05, ** *p* < 0.01, *** *p* < 0.001, and **** *p* < 0.0001 (data were analyzed using an ANOVA); ns, not significant.

All animal experiments were conducted using 7-week-old male C57BL/6 mice (Dooyeol Biotech, Seoul, Republic of Korea). Twenty-five mice were randomly divided into the following groups: control (water only), 2.5% DSS only, DSS plus MBE (100 and 200 mg/kg/day), and MBE only (200 mg/kg/day) (*n* = 5/group). MBE was dissolved in distilled water and administered orally once daily for 3 weeks. For oral administration, we weighed the animals and calculated the volume of the MBE solution required. A gavage needle (gauge 20, length 1–2 inches, curved shape) appropriate for body weight, was inserted into the mouse, directly over the tongue and into the pharynx. For the last seven days (from day 14 to day 21), 2.5% DSS was administered in drinking water ad libitum. The weights of the mice were recorded daily for the last 7 days. At the end of the animal experiments, all mice were anesthetized with tribromoethanol (Avertin) and the colon and blood were immediately harvested. The colon tissue was stored in 4% paraformaldehyde and was subsequently used to prepare paraffin sections for staining. Serum was obtained by centrifuging blood at 1500× *g* for 10 min; it was refrigerated and used for measuring the cytokine level. Hematoxylin and eosin (H&E) and TUNEL-stained colonic tissue sections were used for histological scoring. All the protocols are illustrated in Figure 1A. All protocols involving animals were performed in accordance with the Republic of Korea Institute of Oriental Medicine (KIOM-22-016) and the Guide for the Care and Use of Laboratory Animals (8th edition, 2011) [30].

### 2.2. Cell Culture and Reagents

Human primary intestinal epithelial cells (InEpC) were maintained in a complete human epithelial cell medium kit (Cat# H6621; Cell biologics, Chigago, IL, USA). Human colorectal adenocarcinoma cells (HT29) cells were maintained in roswell park memorial institute (RPMI) (Cat# LM011-60, Welgene, Gyeongsan, Republic of Korea) containing 10% fetal bovine serum (Cat# 12662029, GIBCO, USA), penicillin (100 U/mL), and streptomycin (100 U/mL) (Cat# 10378016, Thermo Scientific, Waltham, MA, USA). MN, HK, and MBE compounds were prepared by dissolving in dimethyl sulfoxide (DMSO) (Sigma-Aldrich, St. Louis, MO, USA).

### 2.3. Cell-Viability Assays

MBE (50 μg/mL to 800 μg/mL), MN (5 μM to 80 μM), and HK (5 μM to 80 μM) were dissolved in DMSO and passed through a filter with a 0.2 µm pore size. Next, 1 × 10^3^ cells were plated in 96-well plates, and MBE and the individual compounds were prepared at different concentrations. After 24 h and 48 h, all samples were incubated in an aqueous non-radioactive cell proliferation solution (MTS) (Cat# G3582, Promega, Madison, WI, USA) for 1 h at 37 °C, and absorbance was recorded at 490 nm.

### 2.4. Immunohistochemistry (IHC)

Immunostaining was performed as previously described, with some modifications. Prior to immunostaining, deparaffinization and hydration were performed in xylene and ethanol in distilled water for 5 min each. Next, the slides were heated in an Antigen Retrieval Solution (Cat# ab93678, Abcam, Cambridge, MA, USA) for 15 min and then incubated with 0.3% hydrogen peroxide for 15 min. The prepared slides were incubated with Sea-Block solution (Cat# 37527, Abcam, Cambridge, MA, USA). The primary antibody was diluted in DAKO diluent solution (Cat# S080983-2; Agilent, Santa Clara, CA, USA) and incubated overnight at 4 °C in a humidified chamber. Slides were incubated with a secondary antibody (Vector Laboratories, Burlingame, CA, USA) for 1 h at room temperature. All samples were incubated with 3,3′-Diaminobenzidine (DAB) solution and counterstained with hematoxylin (Leica, Buffalo Grove, IL, USA). Antibody information is provided in Supplementary Table S1. To evaluate cell death in tissues, terminal deoxynucleotidyl transferase-mediated dUTP nick end labeling (TUNEL) staining was performed. Deparaffinized tissue sections were digested with Proteinase K at 55 °C for 1 h and stained using the TUNEL detection kit (Cat# ab206386, Abcam, Cambridge, MA, USA). The TUNEL assay was performed according to the manufacturer’s instructions. ImageJ version 1.52a software was used to quantify histologic scoring.

### 2.5. Immunoblotting

A total of 1 × 10^6^ cells were plated in 6-well plates, and different concentrations of MBE, MN, and HK were added. Total cell lysates (30 µg) were extracted using mPER buffer (Cat# 78501; Thermo Scientific, Waltham, MA, USA) supplemented with a protease inhibitor cocktail (Cat# 4693116001; Roche, Basel, Switzerland). All antibodies were diluted using HIKARI signal enhancer solution (Cat# 02267-41, NACALAI, Kyoto, Japan). Molecular weight markers (Bio-Rad, Hercules, CA, USA) were loaded onto 4–20% TGX gels (Cat# 4561094, Bio-Rad) and run for 100 min at 100 V. The proteins were then transferred onto a nitrocellulose membrane (Cat#2001, Bio-Rad, CA, USA). The antibodies used are listed in Supplementary Table S1. All approximate molecular weights were decided by comparison with the migration of pre-stained protein standards. A ChemiDoc MP imaging system was used to produce digital images (Bio-Rad, Hercules, CA, USA). The quantitative protein band was performed using ImageJ software (National Institutes of Health, Rockville, MD, USA) and was shown in Supplementary Figures.

### 2.6. Interleukin 6 Cytokine Assay

For quantification of IL-6 in the blood, serum was analyzed using an enzyme-linked immunosorbent assay (ELISA) kit (ab222503, Abcam, Cambridge, UK). Briefly, all blood samples were mixed with the IL-6 antibody in each well and incubated with streptavidin-conjugated horseradish peroxidase for 3 h. Then, all samples reacted with the substrate, and fluorescence intensity was analyzed using an ELISA microplate reader (Synergy HTX Multi-Mode Reader; BioTeK, Winooski, VT, USA).

### 2.7. Sample Preparation and Chemical Profiling of MBE

Powdered MBE was purchased from KOCBiotech (Daejeon, Daejeon, Republic of Korea) and stored in a −20 °C freezer at the Republic of Korea Institute of Oriental Medicine. Dried MBE was extracted using 70% ethanol under reflux for 6 h at 90 °C and lyophilized after filtration. The dried extracts were diluted with methanol for high-performance liquid chromatography (HPLC) analysis. In addition, two standard compounds (MN and HK) dissolved in methanol were analyzed to confirm the bioactive components in MBE. The MBE extracts and individual components were analyzed using Agilent 1260 Infinity LC (Agilent Corporation, Santa Clara, CA, USA) equipped with a Zorbax Eclipse XDB-C18 column (4.6 mm × 50 mm i.d., 5 µm; flow rate 0.3 mL/min) maintained at 20 °C. An HPLC diode array detector was used for detection at 254 nm. Chromatographic data were interpreted using the LabSolutions Multi PDA software. All compounds were purchased from Sigma-Aldrich (St. Louis, MO, USA).

### 2.8. Measurement of ROS Production

Endogenous levels of ROS were determined using a flow cytometer with 2’,7’-dichlorodihydrofluorescein diacetate (DCFDA) (Cat# D399, Invitrogen, Waltham, MA, USA). Briefly, 1 × 10^6^ cells were plated and exposed to different concentrations of MBE, MN, and HK at 37 °C for 4 h. The positive control was treated with the Smac mimetic with z-VAD-fmk (SZ) necroptosis inducer under the same conditions. After staining with 10 μmol/L DCFDA at 37 °C for 30 min, cells were collected and washed five times with phosphate buffered saline (PBS). The fluorescence intensities of cells were tested with a flow cytometer (BD LSRFortessa X-20, Becton-Dickinson, San Jose, CA, USA).

### 2.9. Annexin V-Propidium Iodide Assay

Cell death was assessed using an Annexin V-FITC Analysis Kit (Cat# BD556547, BD, Franklin Lakes, NJ, USA). The annexin V-propidium iodide (PI) assay was performed according to the manufacturer’s instructions. Briefly, InEpC and HT29 cells were plated in 12-well plates at a density of 1 × 10^5^ cells/well and pretreated with or without MBE, MN, or HK for 30 min. The cells were then treated with SZ to induce necroptosis. Cell pellets were resuspended in a 200 μL buffer containing 5 μL annexin V-FITC for 20 min and then incubated with 5 μL propidium iodide for 10 min at room temperature in the dark. After staining, cells were collected and washed five times with PBS. The fluorescence intensities of cells were tested with a flow cytometer (BD LSRFortessa X-20, Becton-Dickinson, San Jose, CA, USA).

### 2.10. Statistical Analysis

Statistical analyses were performed using GraphPad Prism (Version 9.0) software and one-way analysis of variance (ANOVA) (Prism, San Diego, CA, USA). The data are expressed as the mean ± standard error (SE), and the levels of significance were set at * *p* < 0.05, ** *p* < 0.01, *** *p* < 0.001, and **** *p* < 0.0001.

## 3. Results

### 3.1. MBE Ameliorates DSS-Induced Colitis

To investigate the effects of MBE on the treatment of IBD, a mouse model of DSS-induced colitis was established. We created five treatment groups, each containing five mice: control (water only), MBE only (200 mg/kg/day), 2.5% DSS only, 100 mg/kg/day MBE plus DSS, and 200 mg/kg/day MBE plus DSS. For the MBE groups, MBE was administered orally for three weeks, and during the last week, 2.5% DSS was administered to a subset of mice to induce colitis (Figure 1A). After one week, the DSS-only group exhibited significant weight loss of 15–20% compared with the control group (water-only). Interestingly, the MBE-administered groups showed a recovery of DDS-induced weight loss. In particular, a significant (*p* = 0.0361) weight recovery of more than 10% was observed in the 200 mg/kg/day MBE high-concentration group (Figure 1B). We proceeded to measure the length of the colon in the different groups to determine the degree of colitis recovery following MBE treatment. In the group that was administered DSS alone, the colon length was shortened by approximately 40% compared with that in the control group; however, in the groups administered MBE, the length of the colon was restored in a dose-dependent manner. In particular, colon length in the group that was administered 200 mg/kg/day recovered by more than 20% compared with that in the DSS-only group (Figure 1C,D). In addition, expression of the inflammatory cytokine IL-6, previously reported to be upregulated by DSS treatment [31], was significantly reduced in the MBE-administered groups (Figure 1E). These results suggest that MBE can ameliorate IBD and the pharmacological mechanism of its effect can be further elucidated using a DSS-induced colitis model.

### 3.2. MBE Attenuates Necroptosis in the Colons of DSS-Treated Mice

**Figure 2:**
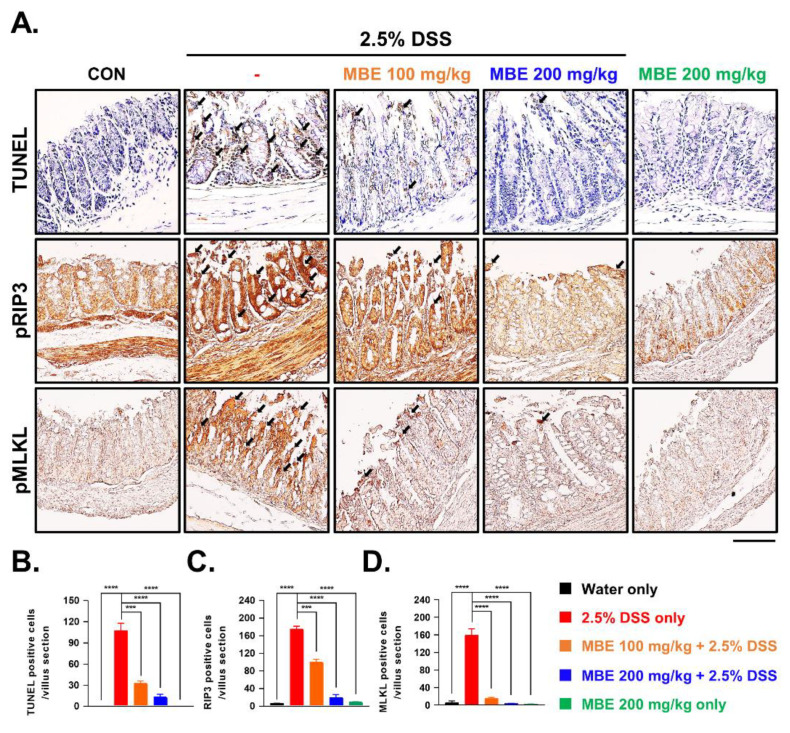
MBE ameliorates DSS-induced damage in the intestinal epithelium. (**A**) Large intestinal sections of mice were stained with TUNEL and pRIP3 and pMLKL antibodies. Colon tissues from mice on day 7 were analyzed using H&E staining and evaluated using histologic score analysis. Scale bar, 100 µm. TUNEL positive cells indicate cell death. Elevated pRIP3 and pMLKL indicate necroptosis. (**B**–**D**) The percentages of TUNEL-, pRIP3-, and pMLKL-positive cells were measured in randomly selected fields and are expressed as numbers of positive cells per field. *n* = 5 for each group. *** *p* < 0.001, and **** *p* < 0.0001 (data were analyzed using an ANOVA).

As mentioned earlier, DSS-induced colitis animal models experience enterocyte death through necroptosis; therefore, suppressing necroptosis may represent a novel treatment avenue for improving IBD. To this end, we first analyzed the death of enterocytes using a TUNEL assay. TUNEL staining was barely detectable in the control group, whereas enterocytes in the DDS-only group were brightly labeled by TUNEL, indicating severe cell death. In contrast, in the MBE-administered groups, the number of TUNEL-positive cells significantly decreased in a dose-dependent manner (Figure 2A,B). To determine whether MBE inhibits DSS-activated necroptosis, the expression levels of the necroptosis markers pRIP3 and pMLKL were compared. The DSS-only group exhibited stronger expression of pRIP3 and pMLKL in enterocytes compared with the control group, and, consistent with our TUNEL results, MBE treatment dramatically reduced the expression of these necroptosis markers (Figure 2A,C,D). Taken together, these findings demonstrate that DSS-induced cell death is associated with necroptosis, which is negatively regulated by MBE, indicating that MBE has the potential to serve as a natural product for treating IBD by preventing necroptosis.

### 3.3. Necroptosis Is Inhibited by MBE in Primary Human Intestinal Epithelial Cells and Colorectal Adenocarcinoma Cells

**Figure 3:**
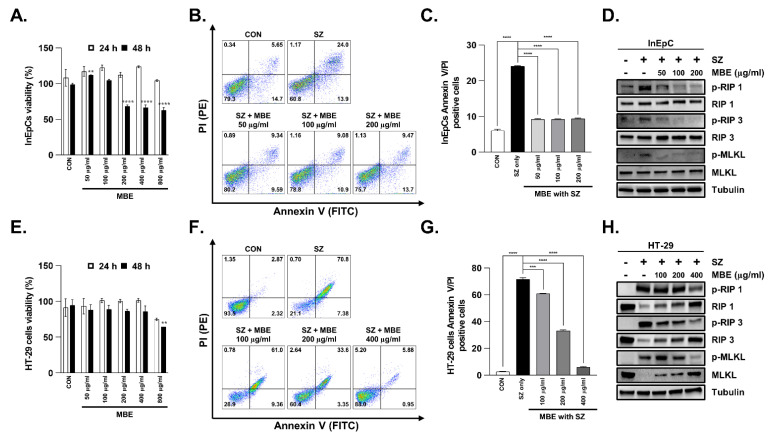
MBE efficiently blocks SZ-induced necroptosis in both InEpCs and HT29 cells. (**A**) Effects of MBE on the viability of InEpC cells treated with the indicated extract concentration for 24 h and 48 h. (**B**) InEpC cells were pretreated with the indicated concentrations of MBE for 30 min before treatment with SZ (Smac mimetic (20 µM) and z−VAD (20 µM)) for 24 h. annexin V/PI was analyzed using flow cytometry. (**C**) The values represent the sum of the percentage of cells in A (PI+ annexin V+; necroptosis). (**D**) Levels of pRIP1, RIP1, pRIP3, RIP3, pMLKL, and MLKL were analyzed using immunoblotting. Tubulin was used as a loading control. (**E**) HT29 cells were treated with the indicated concentrations of MBE for 24 h and 48 h. (**F**) HT29 cells were pretreated with the indicated concentrations of MBE for 30 min before treatment with SZ (Smac mimetic (100 nM) and z−VAD (20 µM)) for 12 h. The expression of annexin V in HT29 cells was induced by SZ and analyzed using flow cytometry. (**G**) The values represent the sum of the percentage of cells in E (PI+ annexin V+; necroptosis). (**H**) Levels of pRIP1, RIP1, pRIP3, RIP3, pMLKL, and MLKL were analyzed using western blotting. Tubulin was used as a loading control. ** *p* < 0.01, *** *p* < 0.001, and **** *p* < 0.0001 (data were analyzed using an ANOVA).

Human primary intestinal epithelial cells (InEpC) and human colorectal adenocarcinoma cells (HT29) were used to verify the biological efficacy of MBE at the cellular level and to further investigate the mechanism of necroptosis inhibition. First, to measure the intracellular toxicity of MBE, cells were treated with different concentrations from 50 to 800 µg/mL. InEpC cells did not exhibit signs of toxicity at any concentration up to 24 h after treatment; however, after 48 h, toxicity was observed at high concentrations (≥200 µg/mL) (Figure 3A). Similarly, HT29 cells showed no toxicity at 24 h, but toxicity was observed at 800 µg/mL after 48 h (Figure 3E). Therefore, the following experiment was performed at an MBE concentration and time that was determined to be non-toxic. Smac mimetic is known to induce apoptosis and necroptosis. The pan-caspase inhibitor z-VAD-fmk (z-VAD) selectively inhibits apoptosis. Therefore, the SZ complex (SZ, Smac mimetic with z-VAD-fmk) was stimulated for 12 h to trigger necroptosis in vitro. We stained the cells with PI and Annexin V to label dying cells and used flow cytometry to assess the level of cell death. SZ increased cell death by approximately 24% compared with the control group (no SZ) in InEpC; however, in the cells pretreated with MBE, necroptosis was inhibited at all concentrations tested from 50 to 200 µg/mL (Figure 3B,C). Approximately 80% of SZ-treated HT29 cells died, and the rate of cell death was reduced by MBE treatment in a dose-dependent manner. In particular, a high concentration of MBE (200 µg/mL) protected against enterocyte death by up to approximately 50% (Figure 3F,G). Next, cells were treated with different concentrations of MBE to investigate the effect of MBE treatment on the expression levels of the necroptosis markers RIP1, RIP3, and MLKL. In both InEpC and HT29 cells without MBE pretreatment, pRIP1, pRIP3, and pMLKL were activated by SZ treatment; however, the levels of all three necroptosis markers were reduced in a dose-dependent manner after pretreatment with MBE for 30 min (Figure 3D,H and Figure S1). Taken together, the protective effect of MBE on enterocyte death was observed both in vitro and in vivo, supporting the efficacy of MBE in ameliorating IBD.

### 3.4. SZ-Induced ROS Production Is Reduced in MBE-Treated Cells

**Figure 4:**
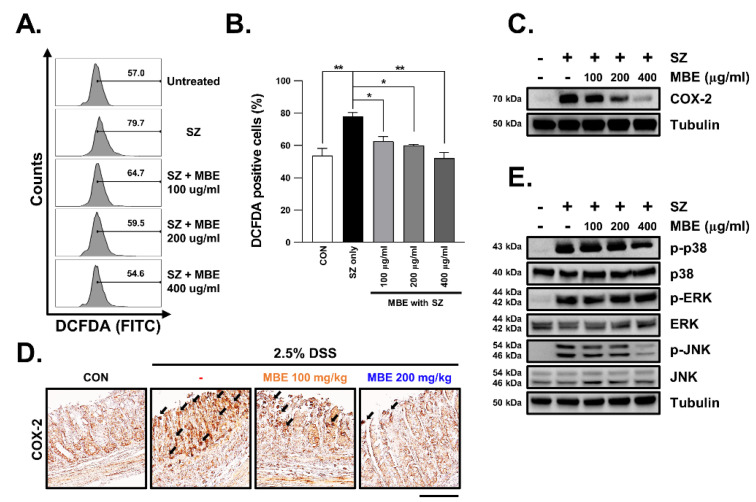
MBE reduces ROS production and the expression of COX2, pp38, and pJNK in SZ-induced necroptosis. (**A**) HT29 cells were pretreated with MBE for 30 min and then treated with SZ (Smac mimetic (100 nM) and z-VAD (20 µM)) for 12 h. Intracellular ROS was detected as DCFDA fluorescence using flow cytometry. (**B**) The number indicates increased DCFDA fluorescence in A. (**C**) Immunoblotting was performed using proteins extracted from HT29 cells. Tubulin was used as an internal control. (**D**) Representative immunohistochemical staining of COX2 in large intestinal sections. Scale bar, 100 µm. (**E**) Levels of pp38, p38, pERK, ERK, pJNK, and JNK analyzed using immunoblotting. Tubulin was used as a loading control. * *p* < 0.05 and ** *p* < 0.01 (data were analyzed using an ANOVA).

A recent study reported that necroptosis caused by inflammation is mediated by ROS [32,33]. To determine whether MBE inhibited necroptosis via ROS signaling or regulated necroptosis independently of ROS signaling, we assessed intracellular ROS production levels. In this way, we found that SZ treatment resulted in an approximately 30% increase in ROS levels. Pretreatment with MBE 30 min prior to SZ treatment resulted in a significant dose-dependent decrease in ROS levels (Figure 4A,B). We next examined the expression of COX2 to confirm that MBE suppressed ROS levels. COX2 is a target gene for ROS and is known to be upregulated in DSS-induced colitis. As expected, COX2 expression was strongly increased in the DSS-only group but was significantly decreased in the MBE-administered groups (Figure 4C and Figure S2). At the cellular level, the increase in COX2 expression caused by SZ treatment was rescued by MBE pretreatment in a dose-dependent manner (Figure 4D). Notably, the JNK and p38-MAPK pathways, which are known to be upstream of COX2 [34,35], are regulated by MBE; therefore, it appears that MBE regulates necroptosis via a ROS-mediated JNK/p38-MAPK/COX2 intracellular signaling mechanism (Figure 4E and Figure S2).

### 3.5. Necroptosis Is Reduced by Honokiol and Magnolol in Primary Human Intestinal Epithelial Cells and Colorectal Adenocarcinoma Cells

**Figure 5:**
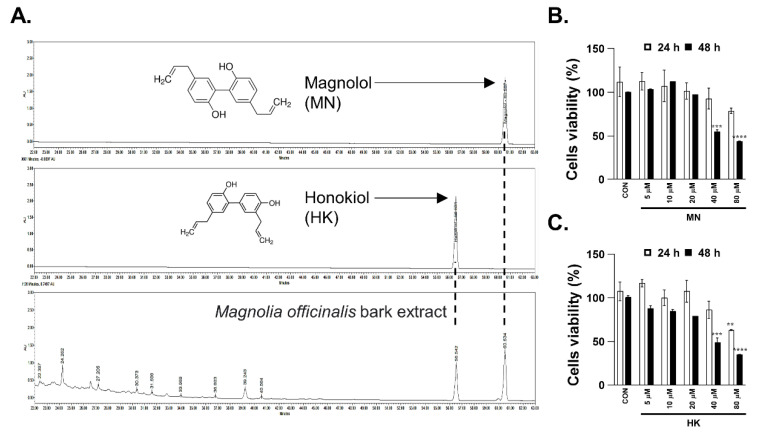
Identification of MBE compounds and cell viability assays. (**A**) HPLC profiles of MBE components. Chemical structures of two standards (Glc = β-D-glucopyranosyl, Rha = α-L-rhamnopyranosyl) and HPLC chromatograms of two standards (1 mg/mL) and a sample extract at a concentration of 20 mg/mL are shown. (**B**–**C**) Viability tests of HT29 cells treated with various concentrations of MN (**B**) and HK (**C**) for 24 h and 48 h. ** *p* < 0.01, *** *p* < 0.001, and **** *p* < 0.0001 (data were analyzed using an ANOVA).

MN and HK are well known as major bioactive compounds in MBE, and so we specifically investigated their effects on necroptosis. First, HPLC was used to verify that the MBE used in this study contained MN and HK (Figure 5A). Cytotoxicity tests indicated no toxicity up to 40 μM after 24 h of treatment for both MN and HK (Figure 5B,C).

**Figure 6:**
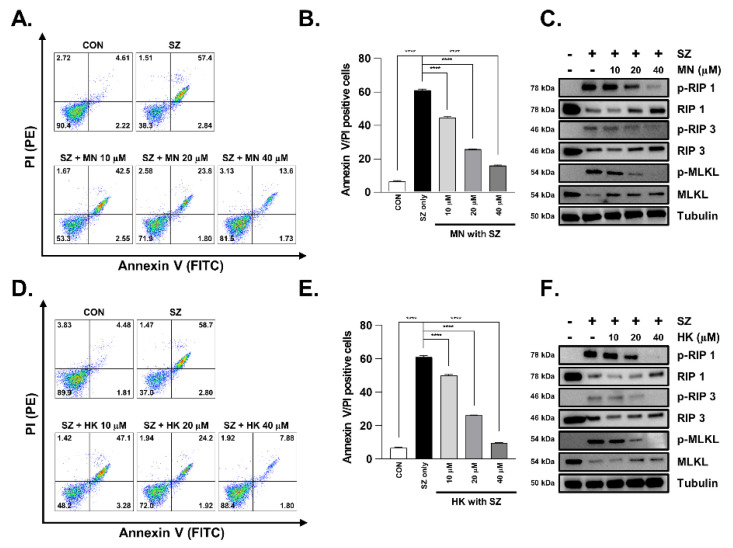
MN and HK inhibit SZ-induced expression of necroptosis proteins in HT29 cells. (**A** and **D**) HT29 cells were pretreated with MN and HK for 30 min before the treatment with SZ (Smac mimetic (100 nM) and z-VAD (20 µM)) for 12 h. (**B**) The values represent the sum of the percentage of cells in A (PI+ annexin V+; necroptosis). (**C** and **F**) Protein levels of pRIP1, RIP1, pRIP3, RIP3, pMLKL, and MLKL were analyzed using western blotting. Tubulin was used as a loading control. (**E**) The values represent the sum of the percentage of cells in D (PI+ annexin V+; necroptosis). **** *p* < 0.0001 (data were analyzed using an ANOVA).

We next assessed cell death using flow cytometry, as was previously assessed for MBE. Cells were pretreated with MN or HK for 30 min and were subsequently treated with SZ for 12 h. Interestingly, both compounds exhibited a significant dose-dependent inhibitory effect on cell death (Figure 6A,B,D,E). Moreover, the levels of pRIP1, pRIP3, and pMLKL, which were increased by SZ treatment, were reduced by MN and HK pretreatment in a dose-dependent manner (Figure 6C,F and Figure S3). Therefore, both MN and HK inhibit necroptosis and may represent natural products for inhibiting enterocyte death in colitis.

### 3.6. SZ-Induced ROS Production Is Reduced by Honokiol and Magnolol through Inhibition of the JNK and p38-MAPK Pathways

**Figure 7:**
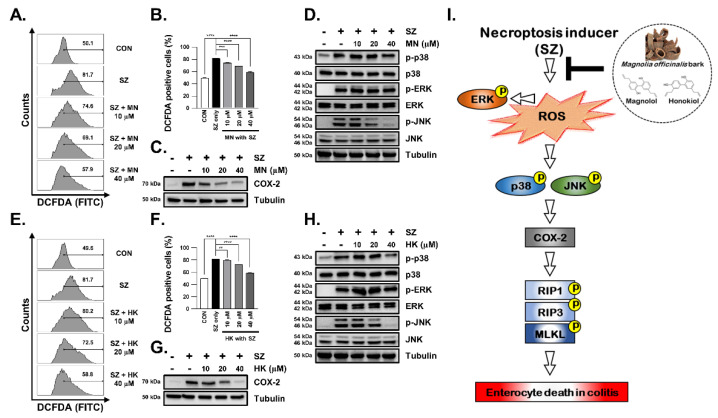
MN and HK attenuate ROS-dependent necroptosis. HT29 cells were pretreated with indicated concentrations of MN (**A**) and HK (**E**) for 30 min before the treatment with SZ (Smac mimetic (100 nM) and z-VAD (20 µM)) for 12 h. Intracellular ROS was detected as DCFDA fluorescence using flow cytometry. (**B**,**F**) The number indicates increased DCFDA fluorescence in (**A**) and (**E**), respectively. (**C**,**G**) The protein expression of COX2 was analyzed using western blotting. Tubulin was used as a loading control. (**D**,**H**) The protein levels of p38, p38, pERK, ERK, pJNK, and JNK were analyzed using western blotting. Tubulin was used as a loading control. ** *p* < 0.01, *** *p* < 0.001 and **** *p* < 0.0001 (data were analyzed using an ANOVA). (**I**) Schematic diagram of the inhibition of the necroptosis pathway by MBE, MN, and HK.

Although MN and HK effectively reduced necroptosis, it is necessary to confirm whether they also inhibit ROS production, similar to MBE. To address this question, ROS production was induced using SZ, and the inhibitory effect of ROS production was investigated by treating cells with MN and HK. Similar to MBE, both HK and MN effectively inhibited ROS production, resulting in a dose-dependent decrease in DCFDA levels (Figure 7A,B,E,F). In addition, COX2 expression was reduced in a dose-dependent manner, and it was suppressed to almost control levels at 40 µM (Figure 7C,G and Figure S4). In addition, the selective regulation of JNK and p38-MAPK is consistent with the results of previous studies using MBE (Figure 7D,H and Figure S4). Therefore, the biological efficacy of MBE can be explained by the complex pharmacological effects of two of its major components, MN and HK. Taken together, our findings strongly support the further investigation of MBE as a novel, natural necroptosis inhibitor with the potential to treat IBD (Figure 7I).

## 4. Discussion

In this study, we demonstrated that MBE may serve as a natural product for regulating necroptosis in a DSS-induced colitis animal model. Using InEpC and HT29 cells, we discovered that the suppression of JNK/p38-MAPK/COX2 signaling by MBE inhibits ROS-mediated necroptosis in vitro. We also showed that MN and HK, two major components of MBE, can exert pharmacological effects by acting as necroptosis inhibitors. Our findings show that MBE can protect against colitis-induced enterocyte death via the inhibition of ROS-mediated necroptosis.

Necroptosis, along with apoptosis, is an important phenomenon involved in intestinal cell damage in IBD. Although the morphological features of necroptotic cell death are similar to those of necrosis, such as cell membrane swelling and disruption of cell membrane integrity, as a form of PCD, it is more often compared with apoptosis. [14,36]. Furthermore, Pierdomenico et al. reported dramatically increased RIPK3 and MLKL expression and significantly decreased caspase-8 expression in the inflammatory tissues of 30 children with ulcerative colitis and 33 with Crohn’s disease [37]; this is consistent with the fact that necroptosis is independent of caspase-8 signaling and dependent on RIPK3 and MLKL. Our findings of increased enterocyte death in the large intestine of mice with DSS-induced colitis concurrent with increases in pRIPK3 and pMLKL are consistent with these previous reports.

Recent studies have reported that intracellular ROS accumulation in response to external inflammatory substances acts as a trigger for necroptosis [38,39]. Herein, we would like to further emphasize that modulating ROS target genes by inhibiting ROS is an important mechanism for alleviating IBD caused by excessive necroptosis. We confirmed that the expression of COX2, a ROS target gene, rapidly increased in the enterocytes of mice with DSS-induced colitis and was effectively downregulated following MBE treatment. COX2 is a representative pro-inflammatory gene induced by an excessive immune response and is known to be regulated by the JNK and p38-MAPK pathways, which, notably, are upstream signals that induce necroptosis. From this perspective, our data support the hypothesis that MBE suppresses necroptosis via the selective control of the COX2, JNK, and p38-MAPK pathways. For the reason that our investigation was limited to COX2 expression, detailed molecular correlation studies between the expression of COX2, other ROS target genes, and necroptosis are needed to validate this mechanism.

Although MBE has been reported to be effective in treating diseases of the digestive system, the underlying molecular mechanisms are not well understood. To address this, we selected two major MBE components, MN and HK, for detailed investigation and found that both components dramatically inhibited necroptosis in vitro. MN and HK are known anticancer agonists. In our study, at high concentrations (800 µg/mL), MBE induced apoptosis in HT29 cells. Therefore, MBE can potentially be used as a natural product to suppress intestinal adenocarcinoma induced by IBD. In contrast, at low concentrations, MBE could be used as a nutraceutical to help maintain enterocyte homeostasis under inflammation. We speculate that their similar effects may be a consequence of similarities in their chemical structures [40]. Notably, the similar efficacies of MN and HK have already been demonstrated in other studies. For example, in the digestive system, Zhang et al. showed the efficacy of both MN and HK in improving GI tract motility [41]. In addition, MN and HK can be used to suppress prednisolone metabolism in the respiratory system and improve the central nervous system as a mechanism to suppress cholinergic deficits [42,43,44]. Nevertheless, our results are the first to report that MN and HK can function as necroptosis inhibitors to suppress colitis. Importantly, since the physicochemical and stability properties of MN and HK differ, it cannot be overlooked that their intracellular pharmacokinetics may differ as well. Thus, to anticipate the synergistic effect of MN and HK, additional research on the suitability of each compound for the target disease is necessary.

## 5. Conclusions

Taken together, our findings demonstrate that MBE and its major bioactive compounds, MN and HK, were effective in alleviating DSS-induced colitis and in mechanistically controlling ROS-mediated necroptosis. Our results highlight the potential of MBE as a disease-targeting drug for anti-colitis therapy.

## Data Availability

Data are contained within the article or Supplementary Materials.

## Supplementary Materials

The following supporting information can be downloaded at: https://www.mdpi.com/article/10.3390/antiox11122435/s1, Table S1: Antibodies for immunoblot and immunohistochemistry, Figure S1: Graph indicates quantification of Figure 3 immunoblot data, Figure S2: Graph indicates quantification of Figure 4 immunoblot data, Figure S3: Graph indicates quantification of Figure 6 immunoblot data, Figure S4: Graph indicates quantification of Figure 7 immunoblot data.
