# Supplementary material for: Magnolia officinalis Bark Extract Prevents Enterocyte Death in a Colitis Mouse Model by Inhibiting ROS-Mediated Necroptosis
Source: Antioxidants (Basel). 2022 Dec 9;11(12):2435. doi: 10.3390/antiox11122435 (PMC9774795; doi:10.3390/antiox11122435)
Supplement: Supplementary file 1 [file antioxidants-11-02435-s001.zip › antioxidants-2068724-supplementary.pdf]

Supporting Information for

Magnolia officinalis bark extract prevents enterocyte death in a colitis mouse model by inhibiting ROS-mediated necroptosis

Kang-In Lee [1], Hye Jin Kim [2], Hyungjun Kim [1], Min-Soo Kim [2], Jung Im Kim [2], and Ki-Sun Park [1,*]

[1] KM Science Research Division, Korea Institute of Oriental Medicine, Daejeon 34054, Republic of Korea.

[2] KM Convergence Research Division, Korea Institute of Oriental Medicine, Daejeon 34054, Republic of Korea.

[*] Corresponding author

E-mail address: kisunpark@kiom.re.kr

Tel.: +82-42-868-9662

Table S1. Antibodies for immunoblot & immunohistochemistry

Antibody	Source	Catalog No.
**RIP1**	Cell signaling	#3493
**p-RIP1**	Cell signaling	#65746
**RIP3**	Cell signaling	#10188
**p-RIP3**	Cell signaling	#93654
**MLKL**	Cell signaling	#14993
**p-MLKL**	Cell signaling	#91689
**p38**	Cell signaling	#9212
**p-p38**	Cell signaling	#9211
**ERK**	Cell signaling	#9102
**p-ERK**	Cell signaling	#4370
**JNK**	Cell signaling	#3708
**p-JNK**	Cell signaling	#4668
**COX-2**	Abcam	Ab52237
**Tubulin**	Abcam	Ab7291

Figure S1. Graph indicates quantification of Figure 3 immunoblot data.

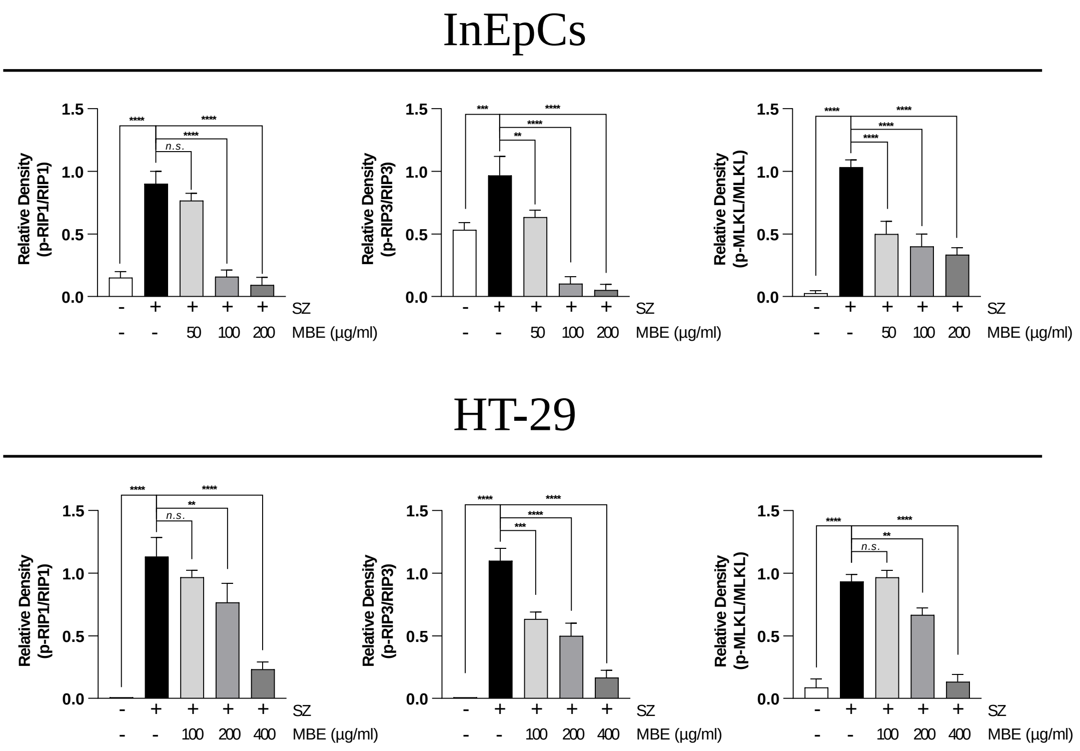

** $P < 0.01$, *** $P < 0.001$, and **** $P < 0.0001$ (data were analyzed using the ANOVA); ns, not significant. (data were analyzed using the ANOVA).

Figure S2. Graph indicates quantification of Figure 4 immunoblot data.

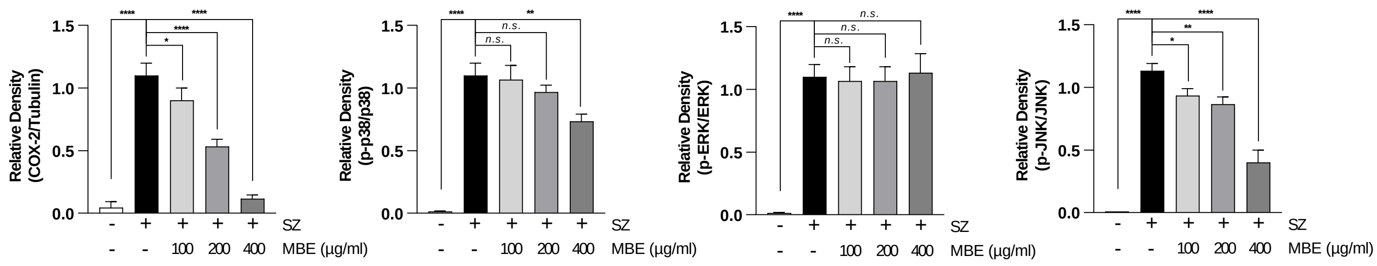

* $P < 0.05$, ** $P < 0.01$, *** $P < 0.001$, and **** $P < 0.0001$ (data were analyzed using the ANOVA); ns, not significant. (data were analyzed using the ANOVA).

Figure S3. Graph indicates quantification of Figure 6 immunoblot data.

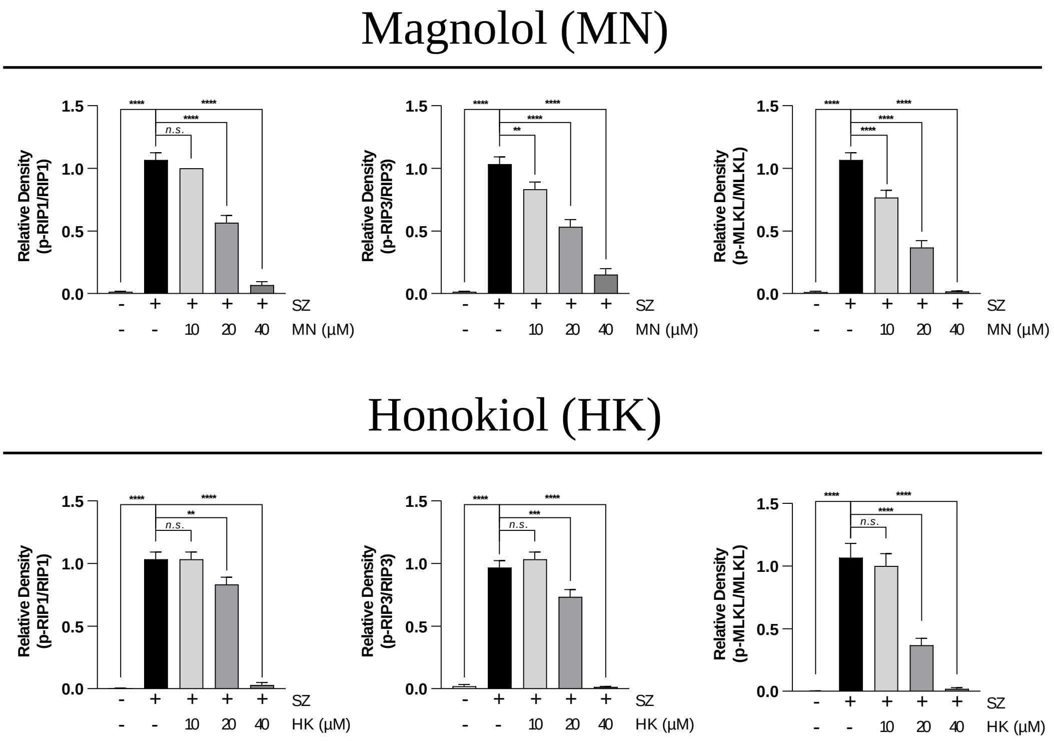

* $P < 0.05$, ** $P < 0.01$, *** $P < 0.001$, and **** $P < 0.0001$ (data were analyzed using the ANOVA); ns, not significant. (data were analyzed using the ANOVA).

Figure S4. Graph indicates quantification of Figure 7 immunoblot data.

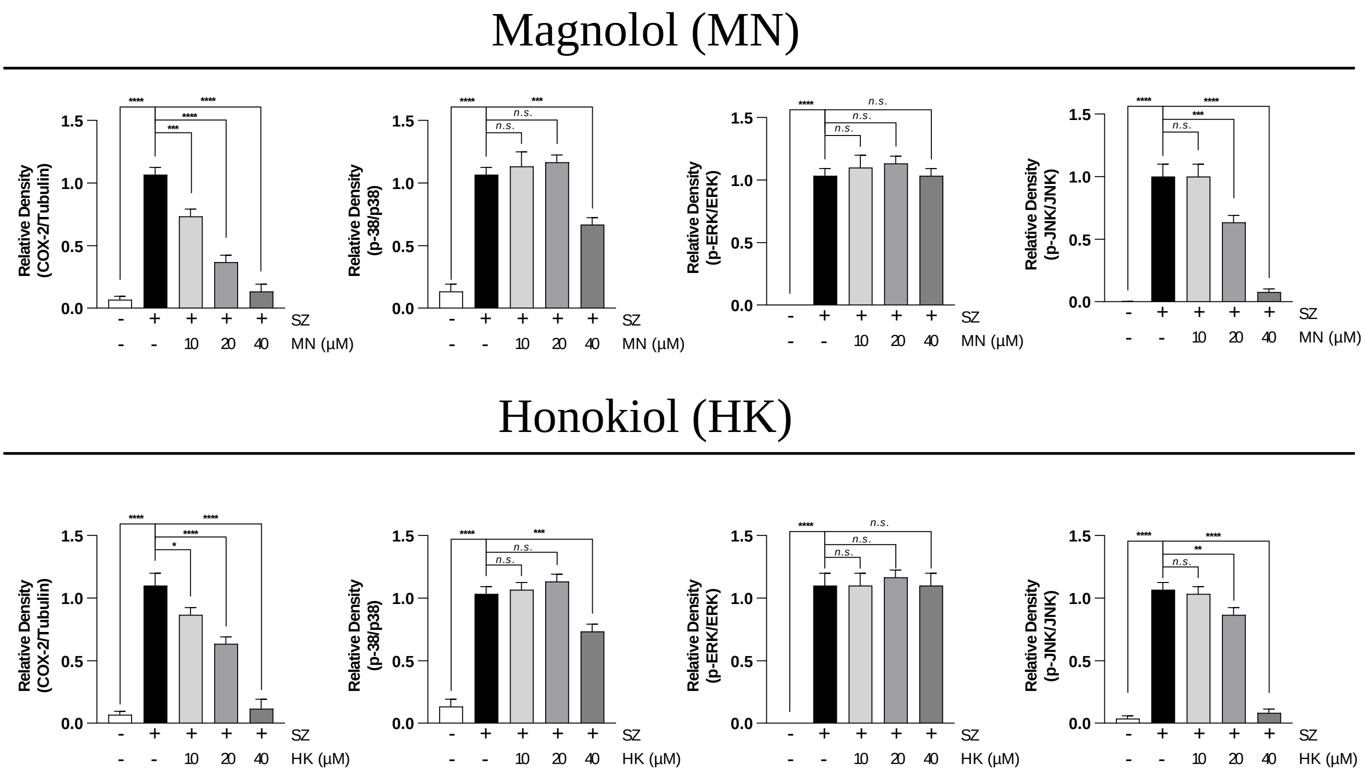

* $P < 0.05$, ** $P < 0.01$, *** $P < 0.001$, and **** $P < 0.0001$ (data were analyzed using the ANOVA); ns, not significant. (data were analyzed using the ANOVA).

Fig 3 D | Fig 3 H

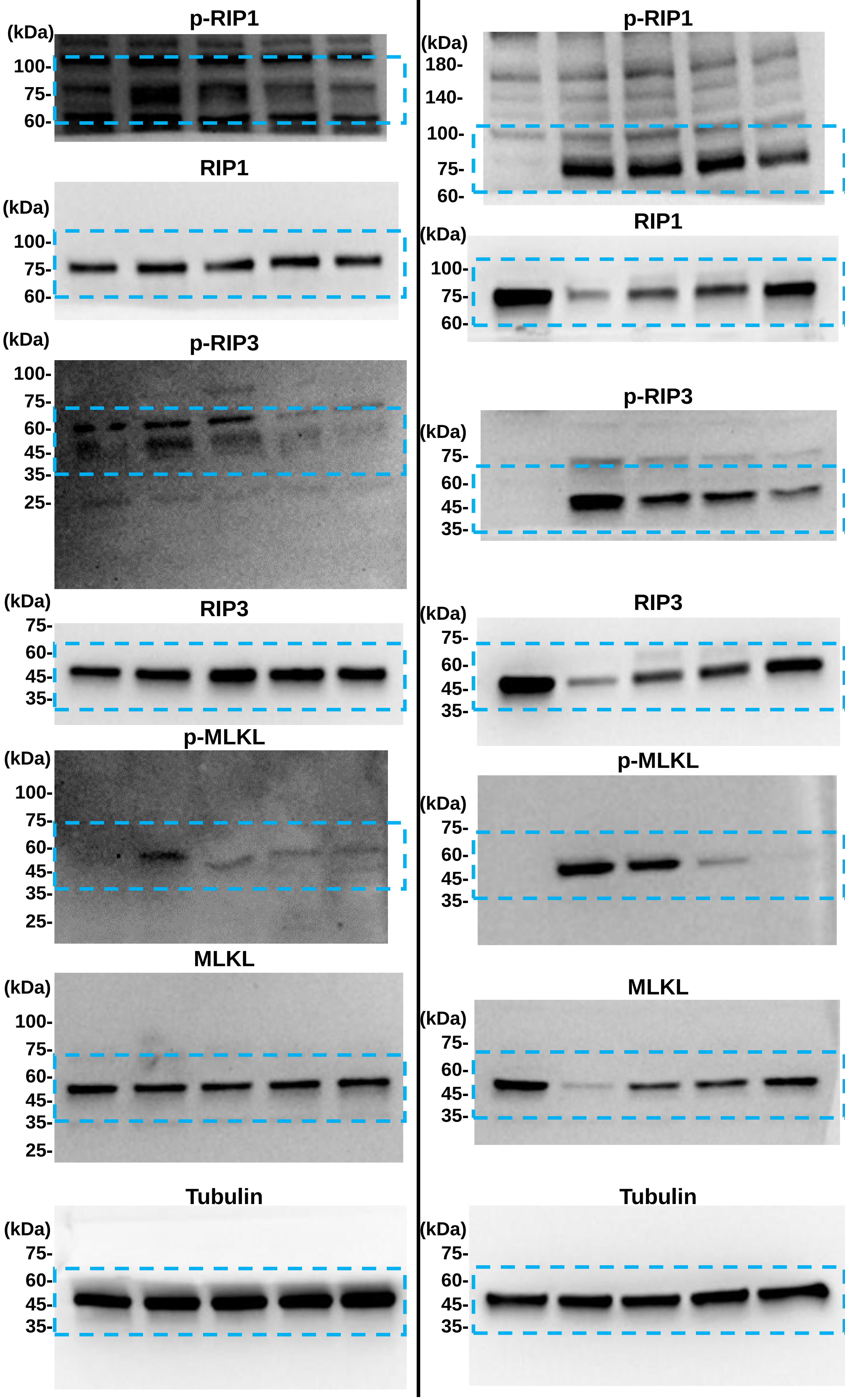

p-RIP1
(kDa)
100-
75-
60-
RIP1
(kDa)
100-
75-
60-
p-RIP3
(kDa)
100-
75-
60-
45-
35-
25-
RIP3
(kDa)
75-
60-
45-
35-
p-MLKL
(kDa)
100-
75-
60-
45-
35-
25-
MLKL
(kDa)
100-
75-
60-
45-
35-
25-
Tubulin
(kDa)
75-
60-
45-
35-
p-RIP1
(kDa)
180-
140-
100-
75-
60-
RIP1
(kDa)
100-
75-
60-
p-RIP3
(kDa)
75-
60-
45-
35-
RIP3
(kDa)
75-
60-
45-
35-
p-MLKL
(kDa)
75-
60-
45-
35-
MLKL
(kDa)
75-
60-
45-
35-
Tubulin
(kDa)
75-
60-
45-
35-

Fig 4 C

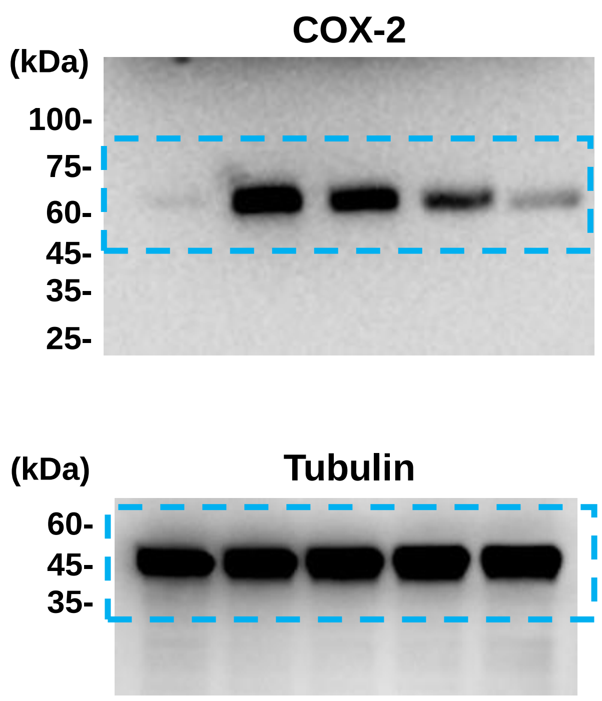

COX-2
(kDa)
100-
75-
60-
45-
35-
25-
Tubulin
(kDa)
60-
45-
35-

Fig 4 E

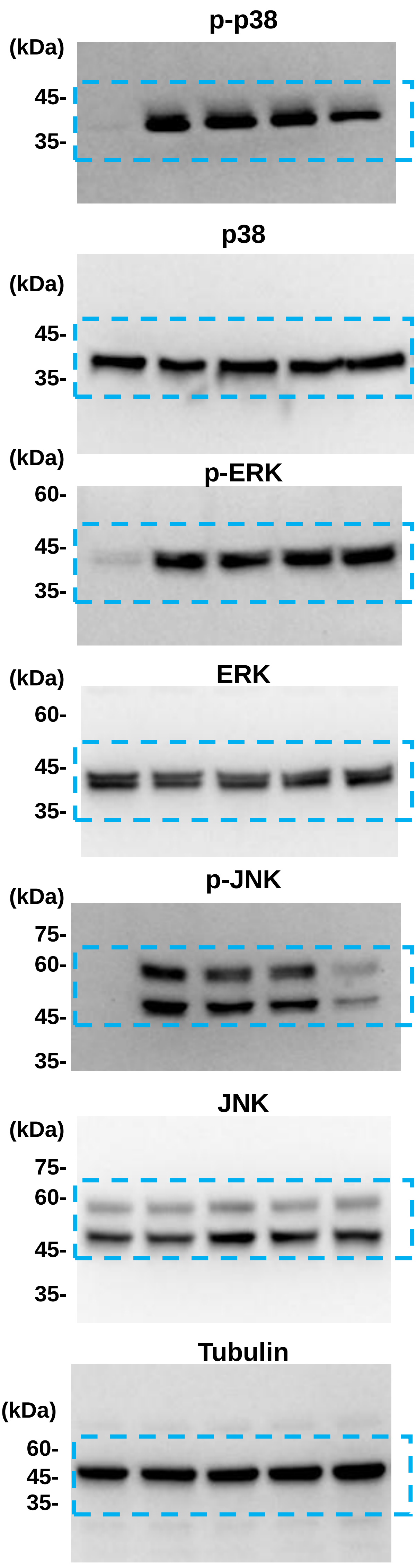

p-p38
(kDa)
45-
35-
p38
(kDa)
45-
35-
p-ERK
(kDa)
60-
45-
35-
ERK
(kDa)
60-
45-
35-
p-JNK
(kDa)
75-
60-
45-
35-
JNK
(kDa)
75-
60-
45-
35-
Tubulin
(kDa)
60-
45-
35-

Fig 6 C

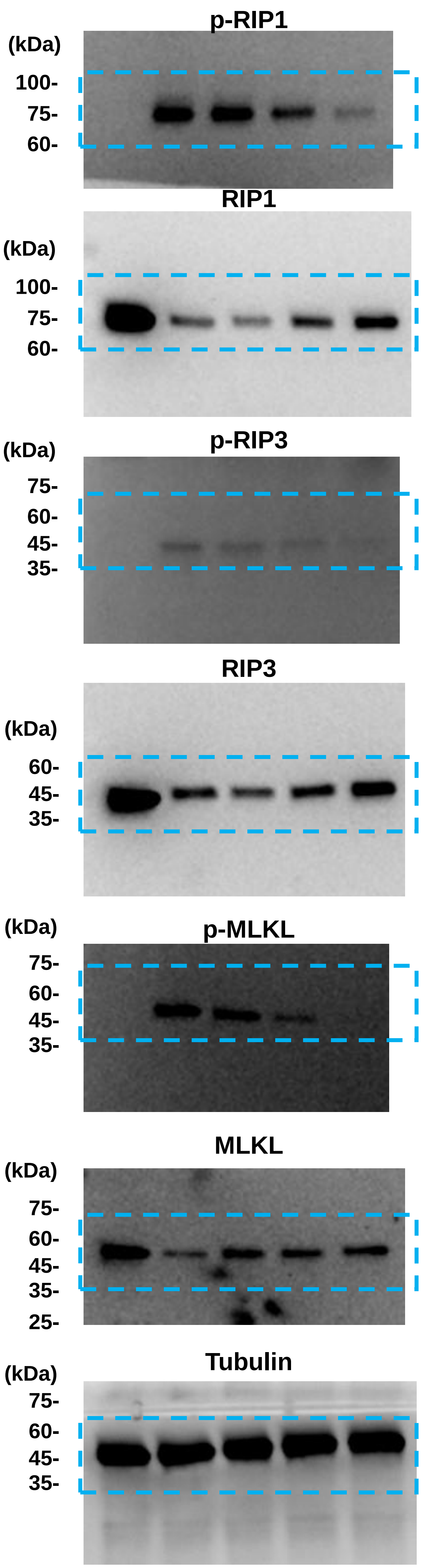
p-RIP1
(kDa)
100-
75-
60-
RIP1
(kDa)
100-
75-
60-
p-RIP3
(kDa)
75-
60-
45-
35-
RIP3
(kDa)
60-
45-
35-
p-MLKL
(kDa)
75-
60-
45-
35-
MLKL
(kDa)
75-
60-
45-
35-
25-
Tubulin
(kDa)
75-
60-
45-
35-

Fig 6 F

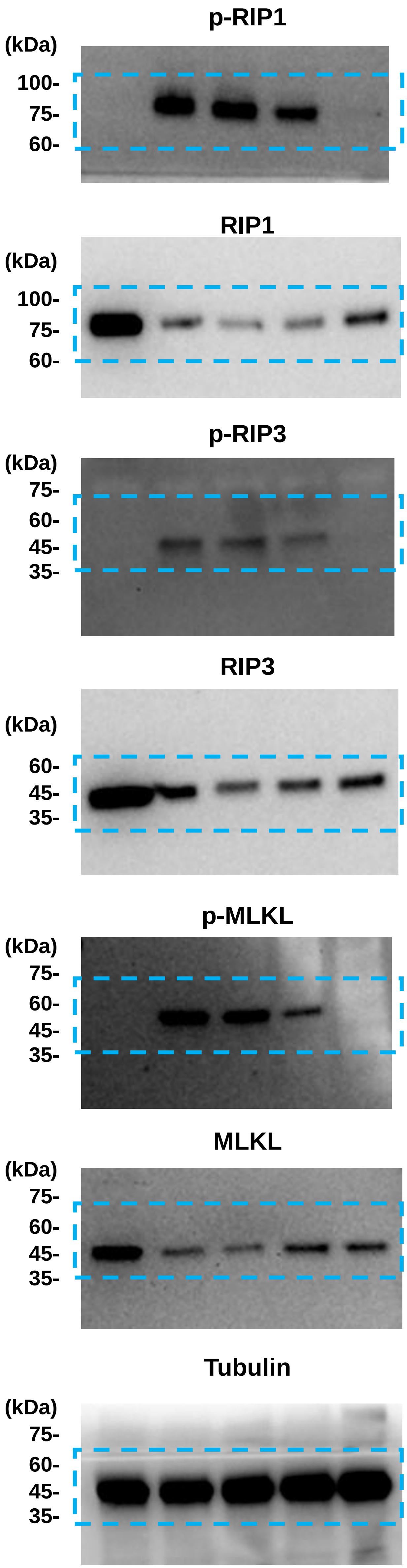
p-RIP1
(kDa)
100-
75-
60-
RIP1
(kDa)
100-
75-
60-
p-RIP3
(kDa)
75-
60-
45-
35-
RIP3
(kDa)
60-
45-
35-
p-MLKL
(kDa)
75-
60-
45-
35-
MLKL
(kDa)
75-
60-
45-
35-
Tubulin
(kDa)
75-
60-
45-
35-

Fig 7 C

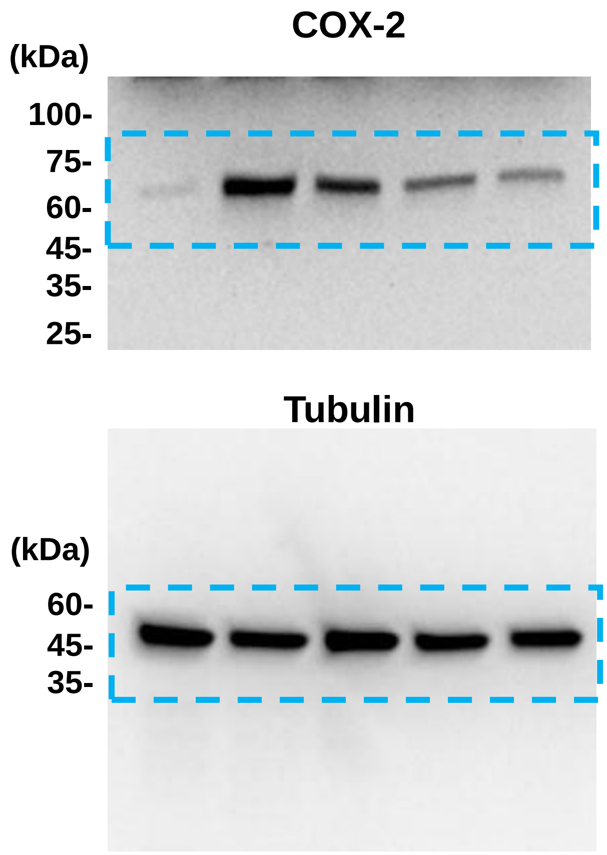
COX-2
(kDa)
100-
75-
60-
45-
35-
25-
Tubulin
(kDa)
60-
45-
35-

Fig 7 D

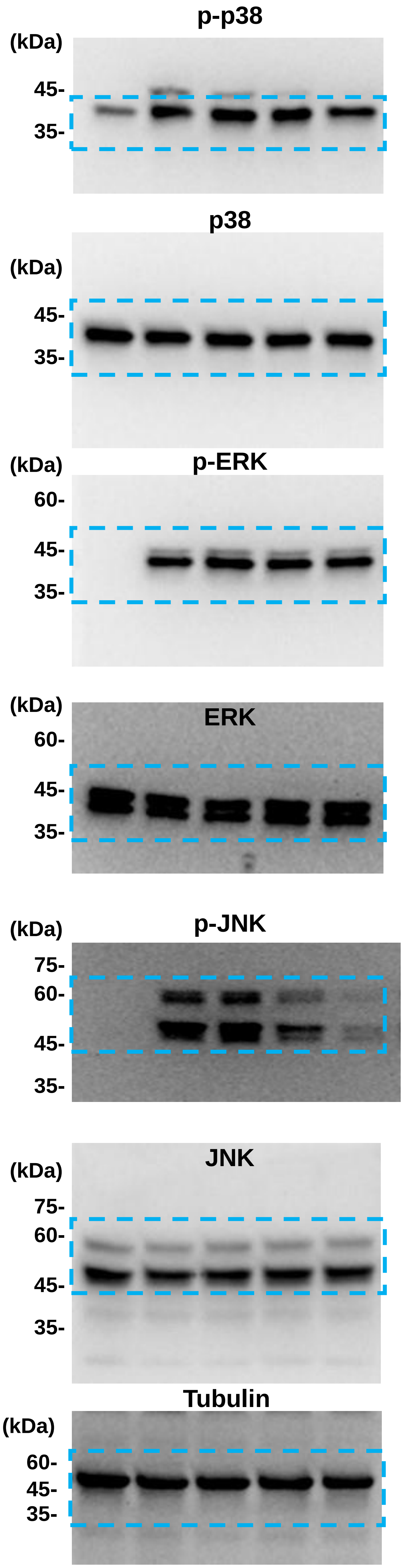
p-p38
(kDa)
45-
35-
p38
(kDa)
45-
35-
p-ERK
(kDa)
60-
45-
35-
ERK
(kDa)
60-
45-
35-
p-JNK
(kDa)
75-
60-
45-
35-
JNK
(kDa)
75-
60-
45-
35-
Tubulin
(kDa)
60-
45-
35-

Fig 7 G

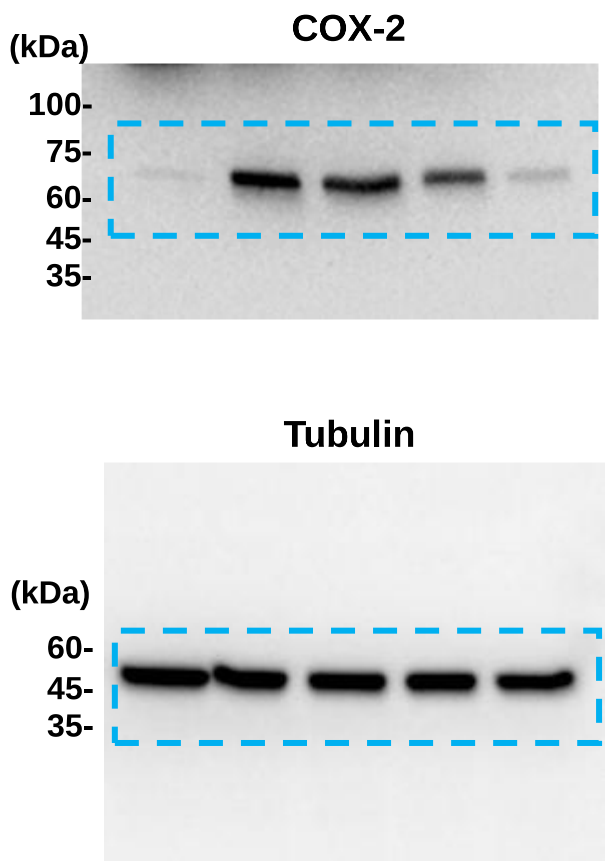
COX-2
(kDa)
100-
75-
60-
45-
35-
Tubulin
(kDa)
60-
45-
35-

Fig 7 H

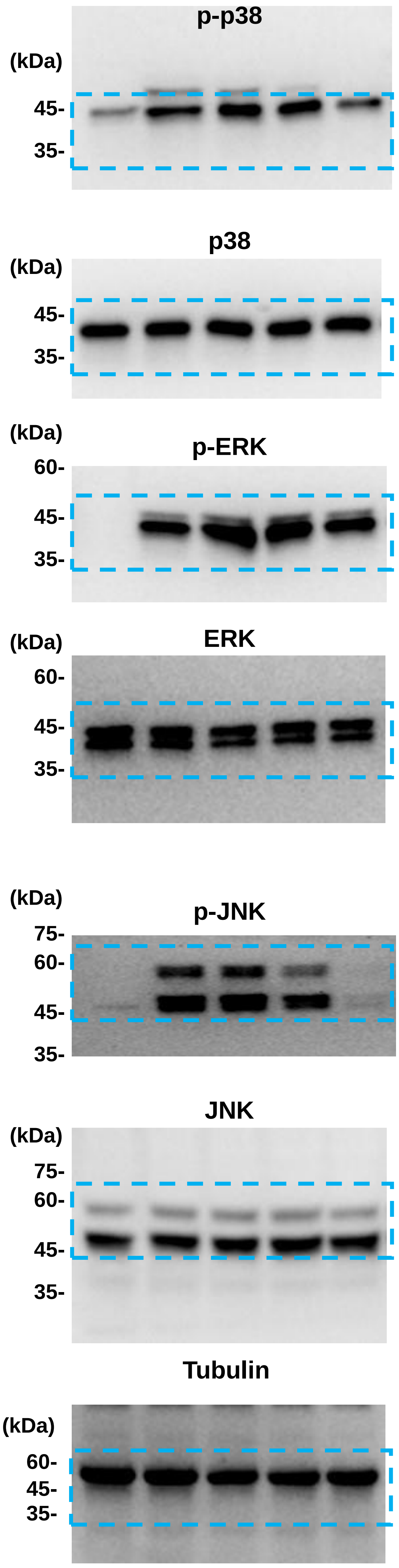
p-p38
(kDa)
45-
35-
p38
(kDa)
45-
35-
(kDa)
p-ERK
60-
45-
35-
(kDa)
ERK
60-
45-
35-
(kDa)
p-JNK
75-
60-
45-
35-
JNK
(kDa)
75-
60-
45-
35-
Tubulin
(kDa)
60-
45-
35-
